# Supplementary material for: Viral GPCR US28 can signal in response to chemokine agonists of nearly unlimited structural degeneracy
Source: eLife. 2018 Jun 8;7:e35850. doi: 10.7554/eLife.35850 (PMC5993540; doi:10.7554/eLife.35850)
Supplement: Figure 1—source data 1. — Exact p-values for chemokines with respect to basal activity using one sample t-test, two-tailed. [file elife-35850-fig1-data1.docx]

| \| Exact p-values from one-sample t-test (two-tailed) 100 nM Chemokine versus basal signaling \| \| \| \| \| \| \| \| \| --- \| --- \| --- \| --- \| --- \| --- \| --- \| --- \| \|  \|  \|  \|  \|  \|  \|  \|  \| \| IP3 Response: \| \|  \| Calcium Response: \| \|  \| Migration Response: \| \| \|  \|  \|  \|  \|  \|  \|  \|  \| \| HEK293 \| <0.0001 \|  \| CX3CL1 \| 0.0002 \|  \| CX3CL1 \| 0.0020 \| \| HEK293 US28 \| 0.9192 \|  \| CCL5 \| <0.0001 \|  \| CCL5 \| <0.0001 \| \| COS-7 US28 \| 0.0002 \|  \| CCL3 \| 0.0251 \|  \| CCL3 \| 0.3311 \| \|  \|  \|  \| vMIP-II \| 0.9079 \|  \| vMIP-II \| 0.5064 \| \|  \|  \|  \| N5F \| 0.0002 \|  \| N5F \| 0.0104 \| \|  \|  \|  \| NVF \| 0.0031 \|  \| NVF \| 0.0026 \| \|  \|  \|  \| deltaUS28 \| 0.2350 \|  \|  \|  \| |
| --- | --- | --- | --- | --- | --- | --- | --- | --- | --- | --- | --- | --- | --- | --- | --- | --- | --- | --- | --- | --- | --- | --- | --- | --- | --- | --- | --- | --- | --- | --- | --- | --- | --- | --- | --- | --- | --- | --- | --- | --- | --- | --- | --- | --- | --- | --- | --- | --- | --- | --- | --- | --- | --- | --- | --- | --- | --- | --- | --- | --- | --- | --- | --- | --- | --- | --- | --- | --- | --- | --- | --- | --- | --- | --- | --- | --- | --- | --- | --- | --- | --- | --- | --- | --- | --- | --- | --- | --- |
