# Supplementary material for: Viral GPCR US28 can signal in response to chemokine agonists of nearly unlimited structural degeneracy
Source: eLife. 2018 Jun 8;7:e35850. doi: 10.7554/eLife.35850 (PMC5993540; doi:10.7554/eLife.35850)
Supplement: Figure 3—source data 1. — Exact p-values for chemokines with respect to the listed reference using one-way ANOVA (Dunnett’s test). [file elife-35850-fig3-data1.docx]

| Exact p-values from one-way ANOVA with Dunnett's correction for 100 nM Chemokine | | | | | | |
| --- | --- | --- | --- | --- | --- | --- |
|  |  |  |  |  |  |  |
| Calcium Response: | | |  | Migration response: | | |
|  |  |  |  |  |  |  |
| WT: QHHGVTK | | reference |  | WT: QHHGVTK | | reference |
| IMHAVNW | | 0.1138 |  | IMHAVNW | | 0.9999 |
| LWSGVSQ | | 0.1069 |  | LWSGVSQ | | 0.6795 |
| VHNLINY | | 0.0062 |  | VHNLINY | | 0.1178 |
| LLPHANY | | 0.0031 |  | LLPHANY | | 0.2608 |
| IRTITNR | | 0.0447 |  | IRTITNR | | 0.3706 |
| IRYSTNK | | 0.9977 |  | IRYSTNK | | 0.0539 |
|  |  |  |  |  |  |  |
| LLPHANY | | reference |  | LLPHANY | | reference |
| ILPHANY | | 0.1368 |  | ILPHANY | | 0.9996 |
| VLPHANY | | 0.5775 |  | VLPHANY | | 0.9545 |
| VRPHVNY | | 0.1486 |  | VRPHVNY | | 0.9195 |
| VRPHINN | | 0.9994 |  | VRPHINN | | 0.5993 |
| LMPGANY | | 0.6617 |  | LMPGANY | | 0.4393 |
| ILPHADY | | 0.9041 |  | ILPHADY | | 0.9209 |
