# Supplementary material for: Viral GPCR US28 can signal in response to chemokine agonists of nearly unlimited structural degeneracy
Source: eLife. 2018 Jun 8;7:e35850. doi: 10.7554/eLife.35850 (PMC5993540; doi:10.7554/eLife.35850)
Supplement: Figure 4—source data 1. [file elife-35850-fig4-data1.docx]

**Figure 4: source data 1. CX3CL1.35 interactions with US28Nb7**

| **"Site 1" (US28 + CX3CL1.35 residues 8-62)** | | | | |
| --- | --- | --- | --- | --- |
| **us28** | **CX3CL1.35** | **distance (Å)** | **type** | **interaction** |
| Asp17 Cγ | Arg47 Nη2 | 3.24 | VDW | sc-sc |
| Asp17 Oδ1 | Arg47 Nη2 | 3.17 | H-bond | sc-sc |
| Asp17 Oδ2 | Gln45 Cδ | 3.27 | VDW | sc-sc |
| Asp17 Oδ2 | Gln45 Nε2 | 2.90 | H-bond | sc-sc |
| Asp17 Oδ2 | Gln45 Oε1 | 2.94 | VDW | sc-sc |
| Asp17 Oδ2 | Arg47 Nη2 | 2.82 | H-bond | sc-sc |
| Glu18 Oε1 | Arg47 Nε | 3.61 | H-bond | sc-sc |
| Glu18 Oε2 | Arg47 Nε | 3.66 | H-bond | sc-sc |
| Glu18 Oε2 | Phe49 Cε1 | 3.29 | VDW | sc-sc |
| Glu18 Oε2 | Phe49 Cζ | 3.61 | VDW | sc-sc |
| Ala20 O | Lys14 O | 3.60 | VDW | mc-mc |
| Ala20 Cβ | Ser13 Oγ | 3.34 | VDW | sc-sc |
| Ala20 Cβ | Lys14 Cβ | 3.68 | VDW | sc-sc |
| Thr21 Cβ | Leu48 O | 3.57 | VDW | sc-mc |
| Thr21 Cγ2 | Leu48 O | 3.31 | VDW | sc-mc |
| Pro22 Cδ | Leu48 C | 3.78 | VDW | sc-mc |
| Pro22 Cδ | Leu48 O | 3.63 | VDW | sc-mc |
| Cys23 N | Thr11 O | 3.17 | H-bond | mc-mc |
| Cys23 O | Thr11 N | 3.45 | H-bond | mc-mc |
| Cys23 O | Thr11 O | 3.77 | VDW | mc-mc |
| Cys23 O | Thr11 Oγ1 | 3.19 | H-bond | mc-sc |
| Val24 Cα | Ala9 O | 3.62 | VDW | mc-mc |
| Phe25 N | Ala9 O | 3.07 | H-bond | mc-mc |
| Phe25 Cε2 | Thr11 Cγ2 | 3.45 | VDW | sc-sc |
| Phe25 Cε2 | Thr11 Oγ1 | 3.16 | VDW | sc-sc |
| Phe25 Cζ | Thr11 Oγ1 | 3.12 | VDW | sc-sc |
| Leu29 Cδ1 | Cys8 O | 3.55 | VDW | sc-mc |
| Leu29 Cδ2 | Cys8 C | 3.71 | VDW | sc-mc |
| Leu29 Cδ2 | Cys8 O | 3.20 | VDW | sc-mc |
| Asp170 O | Gln31 O | 2.98 | VDW | mc-mc |
| Asp170 Cβ | Gln31 O | 3.15 | VDW | sc-mc |
| Asp170 Cγ | Asn30 O | 3.53 | VDW | sc-mc |
| Asp170 Oδ2 | Asn30 O | 2.82 | H-bond | sc-mc |
| Met174 Sδ | Ser33 O | 3.26 | VDW | sc-mc |
| Met174 Cε | Ser33 O | 3.55 | VDW | sc-mc |
| Met174 Cε | Cys34 Cα | 3.60 | VDW | sc-mc |
| Met174 Cε | Cys34 O | 3.79 | VDW | sc-mc |
| Tyr177 Cδ2 | Cys34 O | 3.38 | VDW | sc-mc |
| Tyr177 Cε2 | Cys34 O | 3.35 | VDW | sc-mc |
| Asp178 Oδ1 | Lys36 Cγ | 3.14 | VDW | sc-sc |
| Asp178 Oδ1 | Arg37 N | 3.33 | H-bond | sc-mc |
| Glu266 Oε1 | Thr11 Oγ1 | 3.65 | VDW | sc-sc |
| Lys270 Cε | Cys8 Sγ | 3.78 | VDW | sc-sc |
| Lys270 Nζ | Cys8 Sγ | 2.56 | SCHB | sc-sc |
| Lys270 Nζ | Cys34 Sγ | 2.92 | SCHB | sc-sc |
|  | | | | |
| **"Site 2" (US28 + CX3CL1.35 residues 1-7)** | | | | |
| **us28** | **CX3CL1.35** | **distance (Å)** | **type** | **interaction** |
| Leu29 Cδ2 | Tyr7 Cα | 3.70 | VDW | sc-mc |
| Leu29 Cδ2 | Tyr7 C | 3.36 | VDW | sc-mc |
| Leu29 Cδ2 | Tyr7 O | 3.29 | VDW | sc-mc |
| Ser32 C | Tyr7 Oη | 3.68 | VDW | mc-sc |
| Ser32 O | Tyr7 Oη | 3.58 | VDW | mc-sc |
| Lys33 N | Tyr7 Oη | 3.58 | H-bond | mc-sc |
| Lys33 Cα | Tyr7 Oη | 3.32 | VDW | mc-sc |
| **"Site 2" (US28 + CX3CL1.35 residues 1-7) Continued** | | | | |
| **us28** | **CX3CL1.35** | **distance (Å)** | **type** | **interaction** |
| Thr36 Cβ | Tyr7 Oη | 3.40 | VDW | sc-sc |
| Thr36 Oγ1 | Tyr7 Oη | 2.95 | H-bond | sc-sc |
| Tyr40 Oη | Leu1 Cδ1 | 3.62 | VDW | sc-sc |
| Phe111 Cδ1 | Leu1 N | 3.50 | VDW | sc-mc |
| Phe111 Cε1 | Leu1 N | 3.19 | VDW | sc-mc |
| Phe111 Cε2 | Leu1 C | 3.78 | VDW | sc-mc |
| Phe111 Cε2 | Leu1 O | 3.59 | VDW | sc-mc |
| Phe111 Cε2 | Leu2 Cδ2 | 3.78 | VDW | sc-sc |
| Phe111 Cζ | Leu1 N | 3.46 | VDW | sc-mc |
| Phe111 Cζ | Leu1 C | 3.68 | VDW | sc-mc |
| Phe111 Cζ | Leu2 N | 3.72 | VDW | sc-mc |
| Phe111 Cζ | Leu2 Cγ | 3.80 | VDW | sc-sc |
| Phe111 Cζ | Leu2 Cδ2 | 3.73 | VDW | sc-sc |
| Tyr112 Oη | Pro3 Cδ | 3.49 | VDW | sc-sc |
| Met115 Sδ | Leu2 Cδ2 | 3.65 | VDW | sc-sc |
| Met174 Cε | Asn6 ND2 | 3.42 | VDW | sc-sc |
| Asn189 Cγ | His4 Cδ2 | 3.72 | VDW | sc-sc |
| Asn189 ND2 | His4 Cδ2 | 3.34 | VDW | sc-sc |
| Asn189 ND2 | His4 Nε2 | 3.15 | H-bond | sc-sc |
| Asn189 Oδ1 | His4 Cδ2 | 3.27 | VDW | sc-sc |
| Asn189 Oδ1 | His4 Nε2 | 3.70 | VDW | sc-sc |
| Leu192 Cδ2 | Pro3 Cγ | 3.59 | VDW | sc-sc |
| Tyr244 Cδ2 | Leu2 Cδ1 | 3.72 | VDW | sc-sc |
| Tyr244 Cε2 | Leu2 Cδ1 | 3.52 | VDW | sc-sc |
| Tyr244 Cζ | Leu2 Cβ | 3.51 | VDW | sc-sc |
| Tyr244 Cζ | Leu2 Cδ1 | 3.66 | VDW | sc-sc |
| Tyr244 Oη | Leu2 C | 3.76 | VDW | sc-mc |
| Tyr244 Oη | Leu2 Cβ | 3.17 | VDW | sc-sc |
| Tyr244 Oη | Pro3 N | 3.39 | VDW | sc-mc |
| Tyr244 Oη | Pro3 O | 2.94 | H-bond | sc-mc |
| Tyr244 Oη | Pro3 Cγ | 3.43 | VDW | sc-sc |
| Tyr244 Oη | Pro3 Cδ | 3.25 | VDW | sc-sc |
| Asp251 Oδ2 | His4 Cγ | 3.59 | VDW | sc-sc |
| Asp251 Oδ2 | His4 Cδ2 | 3.64 | VDW | sc-sc |
| Asp251 Oδ2 | His4 ND1 | 3.60 | VDW | sc-sc |
| Asp251 Oδ2 | His4 Cε1 | 3.66 | VDW | sc-sc |
| Asp251 Oδ2 | His4 Nε2 | 3.68 | VDW | sc-sc |
| Leu255 Cδ1 | His4 Cε1 | 3.56 | VDW | sc-sc |
| Lys270 Cε | Asn6 O | 3.24 | VDW | sc-mc |
| Lys270 Nζ | Asn6 O | 3.49 | VDW | sc-mc |
| Leu273 O | Ala5 Cβ | 3.63 | VDW | mc-sc |
| Leu273 Cδ1 | His4 C | 3.76 | VDW | sc-mc |
| Leu273 Cδ1 | His4 O | 3.25 | VDW | sc-mc |
| Leu273 Cδ1 | His4 Cβ | 3.72 | VDW | sc-sc |
| ILE274 Cγ2 | Tyr7 Oη | 3.73 | VDW | sc-sc |
| Glu277 Cδ | Leu2 N | 3.41 | VDW | sc-mc |
| Glu277 Cδ | Ala5 Cβ | 3.67 | VDW | sc-sc |
| Glu277 Oε2 | Leu1 Cα | 3.79 | VDW | sc-mc |
| Glu277 Oε2 | Leu1 C | 3.54 | VDW | sc-mc |
| Glu277 Oε2 | Leu2 N | 2.58 | H-bond | sc-mc |
| Glu277 Oε2 | Leu2 Cα | 3.17 | VDW | sc-mc |
| Glu277 Oε2 | Leu2 C | 3.46 | VDW | sc-mc |
| Glu277 Oε2 | Leu2 O | 3.03 | VDW | sc-mc |
| Glu277 Oε2 | Leu2 Cβ | 3.13 | VDW | sc-sc |
| Glu277 Oε2 | Ala5 Cβ | 3.13 | VDW | sc-sc |
