# Supplementary material for: Viral GPCR US28 can signal in response to chemokine agonists of nearly unlimited structural degeneracy
Source: eLife. 2018 Jun 8;7:e35850. doi: 10.7554/eLife.35850 (PMC5993540; doi:10.7554/eLife.35850)
Supplement: Figure 5—source data 1. [file elife-35850-fig5-data1.docx]

**Figure 5: source data 1. Summary of crystallization and crystallographic statistics.**

|  | US28Nb7 (*apo*) | CX3CL1.35-US28Nb7-Nb B1 |
| --- | --- | --- |
| **Crystallization** |  |  |
| Method | LCP / Glass sandwich | LCP / Glass sandwich |
| Host lipid | Monoolein/cholesterol = 10/1 (w/w) | Monoolein/cholesterol = 10/1 (w/w) |
| Temperature (°C) | 20 | 16-20 |
| Sample Buf. | HBS + 0.02% DDM/0.004% CHS | HBS + 0.02% DDM/0.004% CHS |
| Crystallization Buf. | - 1. M MES pH6.2-6.6   37-41% PEG300  0.1 M ammonium tartrate | 0.1 M MES pH6, 35-39% PEG300  0.05 M lithium sulfate  1% 1,2,3-heptanetriol |
|  |  |  |
| **Data collection** |  |  |
| Beamline | 23-ID-D (APS) | 23-ID-D (APS) |
| No. of crystals | 2 | 2 |
| Wavelength | 1.033 | 1.033 |
| Space group | *P2_1_* | *P2_1_2_1_2* |
| Cell dimensions |  |  |
| *a*, *b*, *c* (Å) | 77.6, 38.3, 99.8 | 47.2, 128.9, 127.7 |
| α, β, γ (°) | 90, 107.4, 90 | 90, 90, 90 |
| Total reflections | 46634 (3879) | 54507 (5609) |
| Unique reflections | 7321 (539) | 10338 (1033) |
| Resolution (Å) | 29.84 - 3.508 (3.633 - 3.508) | 45.37 - 3.500 (3.625 - 3.500) |
| *R*_merge_ | 0.2232 (1.195) | 0.2594 (1.229) |
| *R*_pim_ | 0.09674 (0.5504) | 0.1226 (0.5727) |
| Wilson B-factor | 101.92 | 94.26 |
| *CC*_1/2_ | 0.992 (0.653) | 0.987 (0.491) |
| *I* / σ*I* | 6.91 (1.32) | 5.25 (1.23) |
| Completeness (%) | 97.14 (76.24) | 99.29 (100) |
| Redundancy | 6.4 (5.5) | 5.3 (5.4) |
|  |  |  |
| **Refinement** |  |  |
| Resolution (Å) | 29.84 - 3.508 (3.633 - 3.508) | 45.37 - 3.500 (3.625 - 3.500) |
| No. of reflections | 7129 (539) | 10332 (1033) |
| *R*_work_ | 0.2423 (0.3279) | 0.2514 (0.3256) |
| *R*_free_ | 0.2887 (0.3563) | 0.2958 (0.3177) |
| No. atoms | 3291 | 4907 |
| Protein | 3291 | 4755 |
| Ligands |  | 152 |
| B-factors | 103.83 | 84.72 |
| Protein | 103.83 | 84.57 |
| Ligands |  | 89.30 |
| R.m.s. deviations |  |  |
| Bond lengths (Å) | 0.002 | 0.003 |
| Bond angles (°) | 0.51 | 0.71 |
| Ramachandran plot | 95.67, 4,09, 0.24 | 96.04, 3.80, 0.17 |

* HBS; 10 mM HEPES pH7.2, 150 mM NaCl. Statistics for the highest-resolution shell are shown in parentheses.

Values for Ramachandran plots are presented as favored, allowed, outlier examined by Molprobity(Chen et al., 2010).
